# Supplementary material for: Crisis response during the COVID-19 pandemic: Changes in social contact and social participation of older Americans
Source: PLoS One. 2023 Jul 27;18(7):e0284935. doi: 10.1371/journal.pone.0284935 (PMC10374158; doi:10.1371/journal.pone.0284935)
Supplement: Attachment — Submitted filename: Review PONE-D-22-10689 _9-3-2022.docx [file pone.0284935.s001.docx]

Review Manuscript **Number:** PONE-D-22-10689
**Manuscript Title:** Crisis Response During the COVID-19 Pandemic: Changes in Social Contact and Social Participation of Older Americans in the US.

Thank you for allowing me the opportunity to read this paper and provide comments.

COVID-19 will continue to be a topic we will grapple with for years to come. This is especially relevant as we concern ourselves with the health and well-being of older adults in America, which is the fastest growing segment of our population. COVID-19 has impacted this group the most and in ways that we would not have imagined pre-COVID-19.

Abstract

**Objective:** **Line 35-37** The authors indicated assessment of two aims, 1) changes in social contact and participation, and 2) roles of socio-demographics and health-related factors. It would be helpful if the authors provided their definition of 1) social contact and 2) social participation so that it is clear from the outset what the authors plan to measure. As it written, it seems as these two constructs could be the same. Secondly, when the author indicates the roles of socio-demographics and health-related factors in relations to what. The authors might revise so that the audience clearly understand the points of your study. As I read the document, sometimes this was clear, most times it was not.

**Discussion:** **Line 52-53** The authors must link this promoting digital literacy and providing access to digital platforms better to social contact and social participation. As written, its digital literacy had not been discussed.

**Line 62:** should be commas after, facilities and homes

**Line 63-65:** Reviewer appreciate the definition of social contact and participation here; but recommend it in the abstract as well.

**Line 74-75:** Reviewer appreciate the first mention of digital literacy in relations to social contact; but recommend mentioning this term in the abstract.

**Line 98-102**: Author’s goals as written here seems to be what the authors might convey in their abstract for objective 2. It is clear and provides the reader with what is to be measured and description of change.

**Line 131:** The term eventually seems an odd phrase. As I add the numbers 414 and 288 to 2486 the total is 3188, which would make this a final result. I would suggest revising.

**Line 149-150:** If I am reading this correctly, it appears that no demographic information was asked of participants in Round 10 but the authors went to Round 9 data base and matched the demographics of participants to Round 10. It is unclear how this was done and might be worth a short explanation of this.

**Line 164:** Was there a specific test that NHATS used to determine dementia status, or did NHATS develop its own test.

**Line 204:** The reviewer suggests that when writing the p value, it should be written consistently throughout the document. I some places the “P” is capitalized and in other places it is not. In most research manuscript the p value is not capitalized.

**Line 262**: Authors use the term *lesser social involvement* is this the same as social contact?

**Line 266:** Authors use the term *lower education degrees* is this the same as having a high school degree?

**Line 379-382:** While authors make a great recommendation of what healthcare providers and other social organizations should do, there are limitations that the older adults does not have access to internet, older adults are not digitally literate (unable to operate a computer), and older adults do not have computers in their homes.

**Line 424:** The authors earlier used the term “no dementia” the authors here use the term “cognitively usual”. Reviewer recommends consistent use of terms.

**Line 428-433:** Reviewer believes this makes my final point. The participants in this study were white, highly educated, and more likely have access to technology within their home. This leads to my conclusion, while this study is helpful to determine policy; but how does this help those who are poor, less educated, and limited to access to technology, increase their quality of social contact or social participation. It seems the group that was most prevalent in this study already had a significant advantage and policies related to making cities more “age friendly” will only add to this advantage for this group.
