# Supplementary material for: Crisis response during the COVID-19 pandemic: Changes in social contact and social participation of older Americans
Source: PLoS One. 2023 Jul 27;18(7):e0284935. doi: 10.1371/journal.pone.0284935 (PMC10374158; doi:10.1371/journal.pone.0284935)
Supplement: Attachment — Submitted filename: Rebuttal Letter R2.docx [file pone.0284935.s003.docx]

**PONE-D-22-10689R1**

**EMID:64fa3d01b4287516**

**Crisis Response During the COVID-19 Pandemic: Changes in Social Contact and Social Participation of Older Americans in the US.**

**Response to Reviewers (Minor Revision)**

Dear Dr. Martell,

We appreciate the chance to make further revisions to our paper for the PLOS ONE Journal and would like to express our gratitude for your thoughtful and meticulous feedback. Our team has taken great care to address each comment and we believe that the additional revisions have significantly enhanced the quality of our manuscript. Furthermore, we have carefully checked and fixed any errors in the paper to make sure it follows PLOS ONE's style requirements.

***Additional Editor Comments to the Authors:***

*1) The abstract (under objective) should define participation.  Exactly, participation in what?*

**Response**: Thanks for the advice. We have rewritten the abstract objective as “This study aimed to assess changes in social contact with family, friends and healthcare providers, as well as social participation in working, volunteering, religious services and other organized activities, among older adults during the COVID-19 pandemic while examining the role of pre-COVID sociodemographic characteristics or cognitive and physical limitations in changes in social contact and participation” (Page 3).

Meanwhile, we polished the first research aim in our introduction as “The goals of this study were to investigate: 1) social contact with family, friends and healthcare providers, social participation in working, volunteering, religious services and other organized activities, and their changes during the COVID-19 pandemic among US older adults” (Page 6).

*2) Please clarify informed consent on page 8.  Does the manuscript now explain that the survey administrators did not collect consent?*

**Response**: Thank you for pointing this out. In our ethics statement on page 7, we have stated that “The National Health and Aging Trends Study (NHATS) is sponsored by the National Institute on Aging and is being conducted by John Hopkins University Bloomberg School of Public Health. John Hopkins Bloomberg School of Public Health Institutional Review Board approved this study, and written informed consent was obtained from all the involved participants or their proxy respondents. As a secondary analysis using NHATS data in the public domain and anonymized, this research did not fall within the regulatory definition of research involving human subjects and thus was exempt from institutional board review. As a compatible use with what the participants have agreed to in the original consent form, we did not collect another one.

”

*3) In describing the regression approach, clarify if/that standard errors were adjusted to correct for heteroscedasticity.*

**Response**: Thank you for this suggestion. We have added the suggested content to our Statistical Analyses paragraph on Page 11 as “To correct for heteroscedasticity, we utilized multivariate linear regression models with Huber–White robust standard errors to separately investigate associations …” The regression estimates with Huber–White robust standard errors were presented in Table 2 and Table 3 with updated notes.

*4) Please respond more fully to reviewer comment 15, re: acknowledging the impact of the lack of* *representativeness in the sample impact on your ability to generalize results.  I believe the current claim oversells.  This discussion in the manuscript should include other demographic characteristics (such as race) in addition to education.  If the you wish to claim more generalizability, the manuscript needs to utilize and engage with weighting estimates at a minimum.*

**Response**: We agree that this is a potential limitation of the study, which has been added and polished in our limitation part “Additionally, most participants reported non-Hispanic White race and were relatively well-educated, making them more likely to have access to technology within their home. Thus, the reported experience and challenges of our participants during the COVID-19 period may not represent the experience of older adults who lacked such facile access, which limits the generalizability of the findings.”
